# Low-Friction of ta-C Coatings Paired with Brass and Other Materials under Vacuum and Atmospheric Conditions

**DOI:** 10.3390/ma15072534

**Published:** 2022-03-30

**Authors:** Fabian Härtwig, Lars Lorenz, Stefan Makowski, Matthias Krause, Carsten Habenicht, Andrés Fabián Lasagni

**Affiliations:** 1 Institut für Fertigungstechnik, Technische Universität Dresden, 01069 Dresden, Germany; lars.lorenz@iws.fraunhofer.de (L.L.); andres_fabian.lasagni@tu-dresden.de (A.F.L.); 2 Coating Characterization, Fraunhofer Institute for Material and Beam Technology IWS, 01277 Dresden, Germany; stefan.makowski@iws.fraunhofer.de; 3 Institute of Ion Beam Physics and Materials Research, Helmholtz-Zentrum Dresden-Rossendorf, 01328 Dresden, Germany; matthias.krause@hzdr.de (M.K.); carsten.habenicht@tu-dresden.de (C.H.)

**Keywords:** diamond-like coatings, wear, tribology, carbon, films

## Abstract

Vacuum environments provide challenging conditions for tribological systems. MoS_2_ is one of the materials commonly known to provide low friction for both ambient and vacuum conditions. However, it also exhibits poor wear resistance and low ability to withstand higher contact pressures. In search of wear-resistant alternatives, superhard hydrogen-free tetrahedral amorphous carbon coatings (ta-C) are explored in this study. Although known to have excellent friction and wear properties in ambient atmospheres, their vacuum performance is limited when self-paired and with steel. In this study, the influence of the paired material on the friction behavior of ta-C is studied using counterbodies made from brass, bronze, copper, silicon carbide, and aluminum oxide, as well as from steel and ta-C coatings as reference materials. Brass was found to be the most promising counterbody material and was further tested in direct comparison to steel, as well as in long-term performance experiments. It was shown that the brass/ta-C friction pair exhibits low friction (µ < 0.1) and high wear in the short term, irrespective of ambient pressure, whereas in the long term, the friction coefficient increases due to a change in the wear mechanism. Al_2_O_3_ was identified as another promising sliding partner against ta-C, with a higher friction coefficient than that of brass (µ = 0.3), but considerably lower wear. All other pairings exhibited high friction, high wear, or both.

## 1. Introduction

Carbon can exist in two different covalently bonded crystal structures, which are distinguished by their bonding configuration and their lattice structure. In graphite, the carbon atoms are arranged with trigonal planar configuration in graphene planes (sp^2^ carbon hybridization), which are stacked in an A–B order. In diamond, they are assembled in a tetrahedral configuration (sp^3^ carbon hybridization). Accordingly, the carbon layers in graphite have high internal strength, but they can be easily sheared with respect to each other. In the tetrahedral configuration of diamond, the material exhibits very high strength and hardness in all dimensions [1].

Coatings made from pure carbon can be amorphous, containing a mixture of both hybridization states. If the coating is composed predominantly of sp^3^-bonded C atoms, it is defined as tetrahedral hydrogen-free amorphous carbon (ta-C) [1]. In cases where sp^2^ bonds dominate, the coating is referred to as hydrogen-free amorphous carbon (a-C) [1]. Carbon coatings can also contain a certain amount of hydrogen, denoted as hydrogenated amorphous carbon films (a-C:H or ta-C:H) [1]. 

Coatings made of ta-C have shown to be very effective at reducing friction in non-lubricated contact and in ambient air [2,3]. This behavior is greatly influenced by humidity, the mating material, and the amount and type of surface defects [4,5,6,7]. The friction performance deteriorates significantly when ambient humidity is very low. This is particularly problematic in high-vacuum applications when unlubricated [4,7]. For ta-C in contact with steel in a high vacuum, the only solution may be lubrication [8]. A. Erdemir was able to show the large difference in friction behavior of ta-C coatings depending on their hydrogen content, as well as the extreme importance of moisture for low-friction performance in self-paired ta-C experiments [9]. In 2005, Meunier et al. showed that ta-C coatings paired with 100Cr6 steel also produce very high wear and a high coefficient of friction (COF) under vacuum conditions [10]. In 2012, Konicek et al. demonstrated that the good lubrication of ta-C benefits from a passivation mechanism of “surface dangling bonds by dissociated water vapor”. They were able to corroborate the findings of Erdemir, showing the poor friction performance (COF > 0.6) of self-paired ta-C in low-humidity environments (1% RH) [6]. Previous investigations of ceramic counterbodies, such as aluminum oxide and silicon nitride, on carbon coatings under normal pressure yielded promising results, even at elevated temperatures, where water is desorbed from the surface. It was also shown that the friction behavior strongly depends on the selection of the counterbody material. An example is aluminum oxide, which exhibits lower wear rates, as well as a more constant friction behavior, when compared to silicon nitride and steel [11]. Multilayering soft and hard ta-C coatings may be another way to improve wear performance and mechanical properties in general [12].

To date, dry friction of hydrogen-free carbon coatings against various metals has mostly been studied for metal-doped a-C coatings. When low friction and low wear are observed for these systems, they can often be attributed to the formation of a protective tribolayer [13,14]. The composition of this layer depends on the carbon coating, the counterbody, the atmosphere, and on the temperature [15,16,17,18]. It has been proposed that the coefficient of friction depends on the ease of shearing of these layers [19]. Most importantly, in the absence of oxygen and water, e.g., under vacuum, they cannot form, leading to the adhesion of metal on the coating surface, in turn resulting in very high wear and friction [13,19,20]. Another area of research possibly improving tribological performance is (co)-doping a-C:H coatings with fluor and fluor–sulfur, which has been shown to improve coating lifetime. However, the total lifetime of the coatings remained under 10 min [21]. Doping with silicon up to 17% at.% also seems to improve the coefficient of friction [22]. Although the tribological interaction of metal counterbodies with hydrogen-free a-C coatings has been less studied, interactions comparable to those found for metal counterbodies have been reported by Gharam et al. [23]. From Fourier transform infrared spectroscopy (FTIR) measurements on aluminum alloy counterbodies, the formation of hydrocarbon compounds was observed only under high humidity, whereas the formation of sp² bonds was found only in the absence of water vapor. For titanium alloys against a-C coatings, the formation of tribolayers due to the presence of water has also been shown to significantly reduce the COF [24]. There is even less work published on dry friction of soft metals against ta-C. 

The present work represents the first systematic study of counterbody influence on the friction properties of ta-C under a high vacuum. Different counterbody materials are paired with ta-C coatings in high-vacuum friction experiments. Afterwards, the most promising material combinations are tested under different pressure conditions to explore the stability of the friction and wear behavior. Additionally, uncoated steel discs are tested in the same conditions to elucidate the role of the ta-C coating. Lastly, long-term experiments are performed to explore whether the friction performance remains stable. 

## 2. Materials and Methods

### 2.1. Sample Preparations

Steel discs (100Cr6/1.3505, hardened to 60 HRC, 24 mm diameter, Ra < 0.03 µm) were coated with ta-C using a commercial physical vapor deposition (PVD) chamber (VTD Vakuumtechnik Dresden GmbH, Dresden, Germany) with a plasma-filtered, laser-ignited, pulsed vacuum-arc process [25]. The samples were mounted inside a commercial PVD chamber with an attached Laser-Arc™ module (Fraunhofer IWS, Dresden, Germany) and rotated with a two-axis planetary drive. Before the deposition, the discs were etched with Ar+ ions using a hollow-cathode plasma source. Afterwards, a 100 nm chromium layer was deposited by magnetron sputtering, followed by the Laser-Arc™ carbon coating process. 

The coating thickness was measured by ball crater method. Using a ZHN nanomechanical test system from Zwick/Roell (Ulm, Germany) and the quasi-continuous stiffness method (QCSM), the Young’s modulus and hardness of the coating were measured. They were calculated as described in EN ISO 14577-4:2016, although using a sigmoid fit model for the extrapolation of the coating indentation modulus, as has been previously reported [26]. A Berkovich tip was used with loads of up to 100 mN. Using surface acoustic wave spectroscopy [27], the calculated Young’s Modulus was verified. All ta-C-coated discs were lapped with a diamond slurry to mirror finish. 

The average coating thickness on the discs was 2.1 ± 0.2 μm, and the Young’s modulus of the coatings was 693 ± 18 GPa. Using the Young´s modulus (E), the sp^3^ content (F) was estimated to be 86.6% using F = E/800 GPa, applicable for hydrogen-free amorphous carbon [28].

The used counterbodies were balls, either made from steel, silicon carbide (SiC), copper, bronze (phosphor bronze), aluminum oxide (Al_2_O_3_), or brass (CuZn35). The nominal diameter of the bronze counterbody was 9.525 mm. All other counterbodies had a diameter of 10 mm. In some cases, steel counterbodies were coated with ta-C (thickness 3.7 ± 0.1 μm, Young’s modulus 430 ± 7 GPa) using the same technique as that used for the ta-C coated discs. The ta-C counterbody balls remained rough in the as-deposited state. All counterbody material specifications, including their vendor, are detailed in Table 1. 

### 2.2. Tribological Experiments

For the tribological experiments, a UHVT universal vacuum tribometer (Tetra, Ilmenau, Germany) operating in a ball-on-disc configuration was used. The tribometer was integrated into a vacuum chamber capable of reaching a pressure range from ambient to 10^−9^ mbar. While measuring, the disc was rotated equivalently to a sliding speed of 3 mm/s with a normal force of 5 N. Most experiments were conducted with a sliding distance of 12 m to allow non-disturbed measurement of high-wear material pairs. For further experiments, the sliding distance was increased to 54 m, 67.5 m, and 72 m. 

The initial experiments were carried out in a pressure range of 1 to 7 × 10^−7^ mbar, subsequently referred to as “high vacuum”. On selected material combinations, differing vacuum conditions were applied to further determine the viability at 10^−2^ mbar (“medium vacuum”) and 10^3^ mbar (“ambient air” or “atmospheric condition”). Tribopairs in this study are labelled with the nomenclature [counterbody]/[disc]. 

### 2.3. Evaluation of Results

The coefficient of friction (COF) was calculated by averaging the measured friction coefficient values from the last 2 m of equivalent sliding distance. The steady state was reached in every case after, at most, 6 m. 

The wear tracks and counterbodies were examined with a Zeiss optical microscope (Carl Zeiss AG, Oberkochen, Germany) to study the appearance of possible tribolayers, as well as the wear mechanism and dimensions of the worn area. The counterbody wear volume was estimated by measuring the size of the wear scar. The volume of the missing spherical cap was calculated, assuming a flat wear scar. 

The disc wear-track cross-section area was measured with a contact profilometer (SURFCOM NEX 001 SD-12, Accretech, Coventry, United Kingdom) and averaged on four different positions evenly spaced over the whole track. Then, the disc wear volume was calculated by multiplying the cross section with the circular-wear track length. The specific wear rates of both the disc and counterbody were calculated by dividing the wear volume by the applied force and the sliding distance [29]. The minimum measurable specific wear rate for the disc was estimated by applying the wear-volume measuring routine to several pristine areas next to the wear track. This procedure results in a volume purely stemming from the natural waviness of the surface and represents the minimum measurable wear volume with a given setup of samples and microscope. The minimum wear coefficient was then calculated accordingly from the minimum measurable wear volume. Since the minimal measurable specific wear rate depends on the sliding distance, it therefore depends on the position of the counterbody on the disc for each specific experiment. Depending on the radius, the minimum specific wear rate would range from 2.1 × 10^−7^ mm^3^/(N × m) to 9.9 × 10^−7^ mm^3^/(N × m) for a sliding range of 12 m. For uniformity and simplicity, the minimum detectable wear rate was defined as 1 × 10^−6^ mm^3^/(N × m) for all measurements with a 12 m sliding range. For experiments with increased sliding range, the minimum detectable wear rate was calculated similarly and defined uniformly at 3 × 10^−7^ mm^3^/(N × m). For the specific wear rate of the counterbody, there is no limit to the measurability since a wear scar diameter can always be measured. 

To calculate the contact pressure at the end of the test, it was assumed that the visible worn-out wear-scar area on the counterbody was equivalent with the contact area between the disc and counterbody. The contact pressure was calculated by dividing the applied normal force by the contact area. 

In addition, Raman spectroscopy was performed ex situ on the wear tracks of selected samples and on the counterbody wear scars. A fiber-coupled micro-Raman setup based on an iHR 550 spectrograph and an LN2-cooled CCD detector was used (Horiba, Oberursel, Germany). For excitation, a laser wavelength of 532 nm and a laser spot power of <1 mW were applied. The diameter of the laser spot on the specimen was 5 µm. To compare the changes in the material composition before and after the tribological tests, measurements of neighboring pristine areas on the discs and counterbodies were made. 

## 3. Results

### 3.1. Screening of Counterbodies in High Vacuum

**Figure 1:**
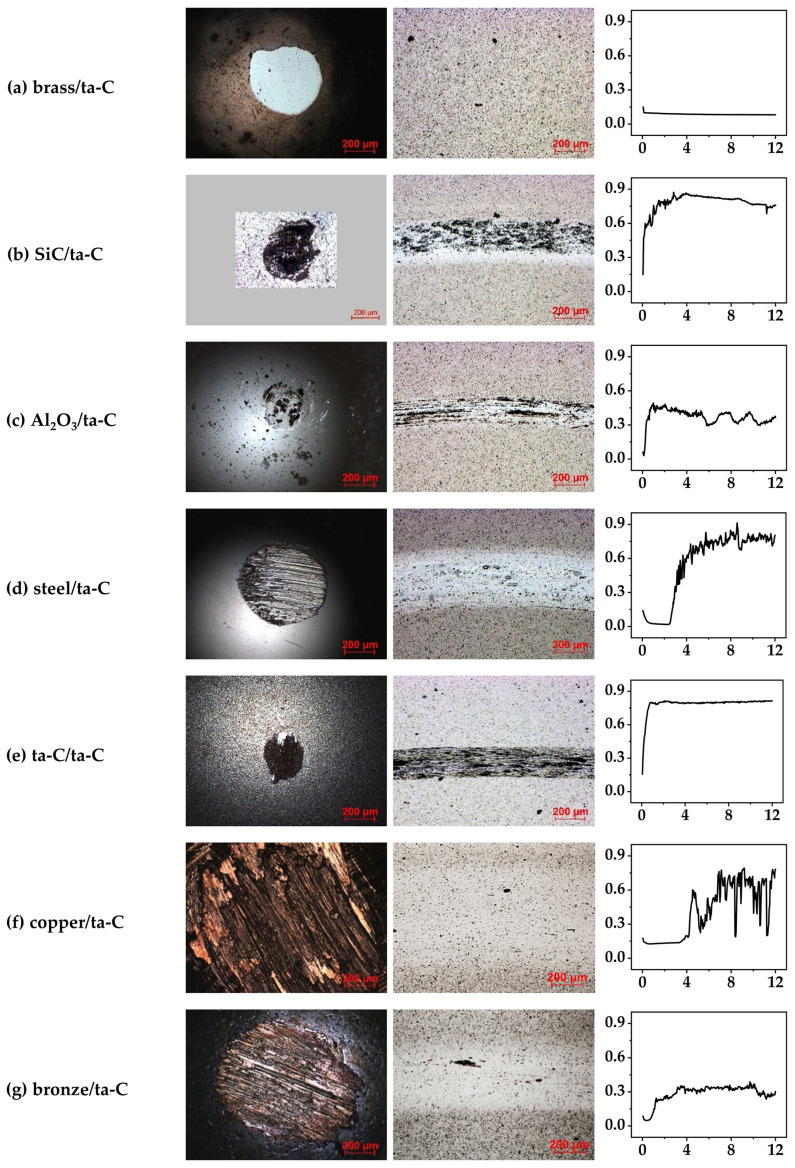
Light microscope images of wear on counterbody (**left**) and disc (**middle**) are shown for various counterbodies: (**a**–**g**) all paired with a ta-C disc under high vacuum. Friction coefficient diagrams are shown (**right**), with friction coefficient on the *y*-axis and sliding distance/m on the *x*-axis. Third-body films are visible in (**b**,**c**,**e**), attributed to transfer of carbon material from the coated disc to the counterbody. Pressures/mbar: brass/ta-C: 1.1 × 10^−7^; SiC/ta-C: 6.7 × 10^−7^; Al_2_O_3_/ta-C: 3.3 × 10^−7^; steel/ta-C: 2.0 × 10^−7^; ta-C/ta-C: 4.7 × 10^−7^; copper/ta-C: 3.6 × 10^−7^; bronze/ta-C: 3.6 × 10^−7^.

**Figure 2:**
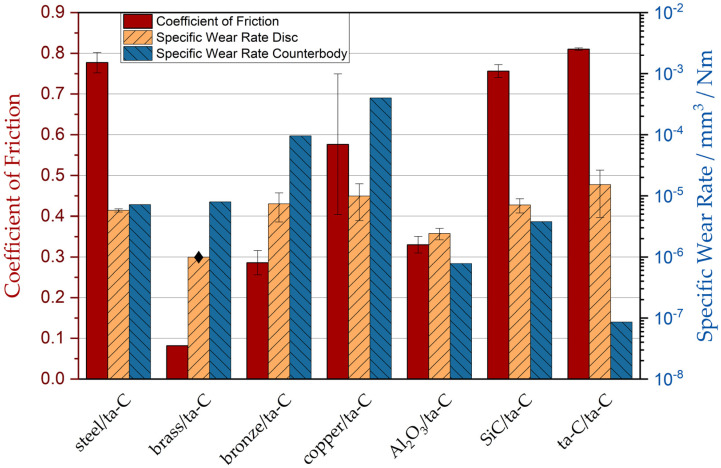
Comparison of steady-state friction coefficients and specific wear rates of discs and counterbodies for different counterbody materials tested against a ta-C surface at a pressure of 1 to 7 × 10^−7^ mbar and 12 m sliding distance. No wear could be found on the disc in the brass/ta-C experiment; therefore, the minimum measurable wear rate is reported and marked with a diamond shape. Pressures/mbar: brass/ta-C: 1.1 × 10^−7^; SiC/ta-C: 6.7 × 10^−7^; Al_2_O_3_/ta-C: 3.3 × 10^−7^; steel/ta-C: 2.0 × 10^−7^; ta-C/ta-C: 4.7 × 10^−7^; copper/ta-C: 3.6 × 10^−7^; bronze/ta-C: 3.6 × 10^−7^.

To study the influence of the counterbody material on the tribological behavior with the ta-C coated discs, wear experiments were conducted using a selection of different materials. All experiments were performed under high vacuum. A strong influence of the counterbody material on friction and wear was observed. Wear tracks of discs and counterbodies for all pairings, as well as friction diagrams, are shown in Figure 1. Friction coefficient, specific counterbody, and disc wear rate are compared in Figure 2.

The optical appearance of the wear tracks and counterbody wear scars depends significantly on the friction pairing (Figure 1). After the experiment with brass/ta-C (Figure 1a), no wear was visible on the ta-C disc, but considerable wear was found on the brass counterbody. Furthermore, no third-body film was visible on either disc or counterbody. 

The combination of brass/ta-C resulted in a very low and stable COF of 0.08 after a very short running-in phase. Brass seems to display a significant advantage over all other tested materials by exhibiting low friction in combination with indiscernible disc wear but high counterbody wear (8.0 × 10^−6^ mm^3^/(N × m)).

The SiC/ta-C pair developed a clearly visible tribofilm on both the counterbody and disc (Figure 1b). The friction coefficient was very high (0.76), whereas the specific counterbody wear rate and the specific disc wear rate were average ([3.8 × 10^−6^ and 7.1 × 10^−6^] mm^3^/(N × m)). 

In the experiment with Al_2_O_3_/ta-C (Figure 1c), a tribofilm formed on both the counterbody and disc, although the film on the counterbody was less pronounced. The friction coefficient, as well as the specific counterbody wear rate and the specific disc wear rate, was relatively low in comparison to the other material combinations (COF = 0.33, [7.8 × 10^−7^ and 2.4 × 10^−6^] mm^3^/(N × m)).

The steel/ta-C pair did not develop a tribofilm (Figure 1d). As in the experiment with SiC/ta-C, the friction coefficient was very high (0.78), with average wear on the counterbody and disc ([7.2 × 10^−6^ and 5.8 × 10^−6^] mm^3^/(N × m)).

In the case of the ta-C/ta-C system, a very pronounced tribofilm was visible, as denoted in the light microscopy pictures in Figure 1e. Whereas the friction coefficient and the specific disc wear rate were the highest of all tested material pairings (0.81 and 1.5 × 10^−5^ mm^3^/(N × m), respectively), the specific counterbody wear rate was the lowest of all pairings (8.6 × 10^−8^ mm^3^/(N × m)).

The copper/ta-C pair (Figure 1f) showed no signs of a tribofilm. The measured COF was high (0.58) but not as high as that of the ta-C/ta-C, steel/ta-C, and SiC/ta-C material combinations. The calculated specific counterbody wear rate was the highest, and the specific disc wear rate was the third highest of all tested materials ([4.0 × 10^−4^ and 9.9 × 10^−6^] mm^3^/(N × m)).

In the experiment with bronze/ta-C (Figure 1g), no tribofilm was developed. Whereas the friction coefficient was relatively low (0.29), the specific counterbody wear rate was the second highest, and the disc wear was the third highest in comparison to the other material pairings ([9.6 × 10^−5^ and 7.4 × 10^−6^] mm^3^/(N × m)).

From the results shown in Figure 1 and Figure 2, it is apparent that wear and friction do not necessarily correlate [30], and the reported values can change in the order of at least one magnitude depending on the material pair. There is no clear difference between the behavior of metallic and ceramic counterbodies. Rather, each friction pairing develops its specific response under the applied high-vacuum conditions. The pairing of brass with ta-C showed the best performance compared to all other tested counterbody materials, followed by bronze and Al_2_O_3_. All other counterbody pairings resulted in unfavorably high friction coefficients.

### 3.2. COF Evolution for the ta-C/Brass System in Vacuum and Atmospheric Conditions 

**Figure 3:**
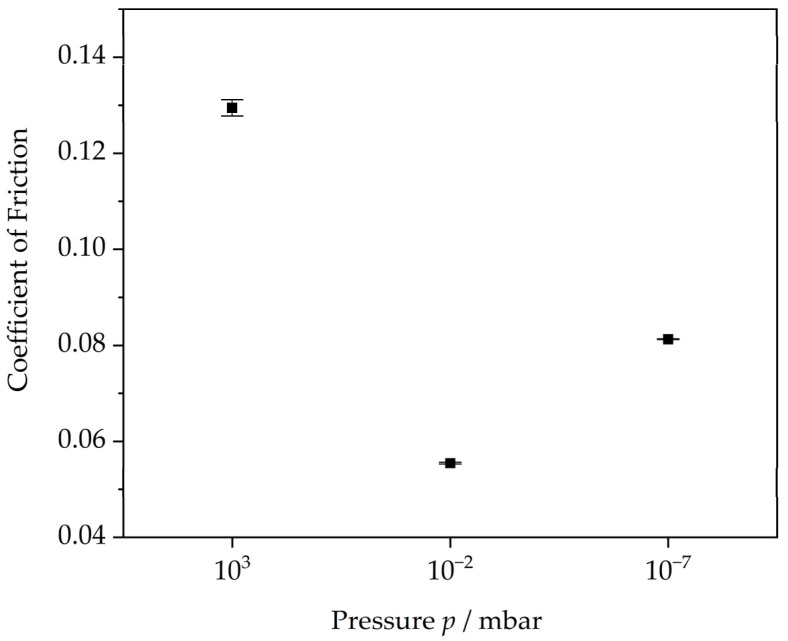
Comparison of steady-state friction coefficient of the brass/ta-C system at different pressures (for 12 m sliding distance).

Since the brass/ta-C pair showed the lowest friction combined with no coating wear, further experiments were performed in ambient air (p = 10^3^ mbar) and medium vacuum (p = 10^−2^ mbar). The steady-state friction data are shown in Figure 3.

The highest friction of the brass/ta-C system arises in ambient air, with a COF of 0.129. In the cases of high vacuum and medium vacuum, the COF was 0.081 and 0.055, respectively. The friction of the brass/ta-C system is thereby influenced by pressure, although no clear linear dependency is observed. Nonetheless, friction coefficients for unlubricated systems up to 0.2 can be considered low from the point of application, which was demonstrated over a wide range of pressures for the brass/ta-C system.

#### 3.2.1. Wear Behavior of the Brass/ta-C Pairing

To study the mechanism for the distinctive wear behavior of the brass/ta-C pairing, Raman measurements were taken on both the disc and the counterbody. Table 2 shows optical microscope images of both ta-C disc wear tracks and counterbody wear scars with their corresponding Raman signals inside and outside the wear track.

On the discs, a wear track on the ta-C coating was only barely visible after the experiment at a pressure of 10^3^ mbar and 10^−2^ mbar and not visible after the high-vacuum experiment (Table 2), all corresponding to less than measurable wear. 

On the brass counterbody, the size and color of the wear scars changed significantly, showing different behaviors depending on the pressure used. The damaged areas were small and dark-colored for 10^3^ mbar, small and light-colored for 10^−2^ mbar, and large and shiny for 10^−7^ mbar. Raman spectroscopy measurements performed inside and outside the worn area were conducted to study the presence and structural changes of the carbon phase. 

Characteristic ta-C Raman spectra are found on the ta-C coated disc in all cases and consist of a slightly asymmetric G band at ~1600 cm^−1^ [31]. No D band around 1350 cm^−1^ is present in the as-deposited films, indicating a fully amorphous structure and the absence of sp^2^-C-clusters, as it is typical for sp^3^-rich and hard coatings. It is noteworthy that the recorded spectra are identical inside and outside the wear track, proving that no structural changes took place in the probed volume during tribological sliding. In the case of the system tested at 10^−7^ mbar, no spectrum is shown, since the wear track could not be accurately identified on the disc. However, given the low shear stress induced by sliding and the analogy to medium-vacuum and atmosphere conditions, an unchanged ta-C structure is assumed as well. It should be noted that no luminescence background was observed in the Raman spectra of the ta-C wear tracks, which could indicate material transfer from the Cu-rich brass counterbody. 

On the counterbody, wear scar spectra differ according to pressure used. For both atmospheric conditions and high vacuum, the Raman signal consists of a broad luminescence background inside and outside the contact area, which is typical for metallic copper. This suggests that no carbon deposit formed in either case, despite the dark-colored transfer layer visible in atmospheric conditions. 

In contrast, a carbon Raman signal with a D and G peak was superimposed on the Cu-based luminescence after the 10^−2^ mbar experiment. The two carbon lines were particularly strong (as visible in Table 2) in the front edge areas of the contact area and almost hidden by the luminescence in other areas. Nevertheless, the detection of narrow D and G lines indicates the formation of a transfer layer whose structure is clearly different from that of the ta-C carbon source on the disc. Tentatively, this structure is attributed to sp^2^-C clusters, since the absence of the 2D line indicates that no ordered graphenic structures were formed.

#### 3.2.2. Comparison with Steel

To study whether this low-friction phenomenon of brass/ta-C in low pressure originates only from the brass counterbody, the ta-C coating alone, or the combination thereof, further experiments with brass/steel and steel/ta-C pairs were performed. 

**Figure 4:**
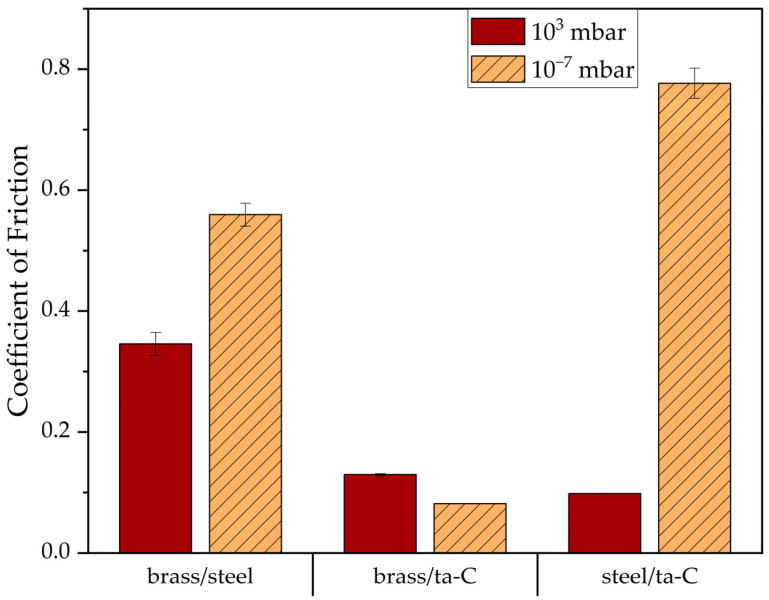
Steady-state friction coefficient of brass/steel, brass/ta-C, and steel/ta-C pairs at 10^3^ mbar and 10^−7^ mbar. High-vacuum pressure/mbar: brass/steel: 5.0 × 10^−7^; brass/ta-C: 1.1 × 10^−7^; steel/ta-C: 2.0 × 10^−7^.

As visible in Figure 4, the pairing of brass/steel exhibited a medium friction coefficient in atmospheric conditions (COF = 0.35) and a moderately high friction coefficient (COF = 0.56) under high vacuum. The brass/ta-C pair showed very low friction in both normal air (COF = 0.13) and under high vacuum (COF = 0.08). The steel/ta-C pair exhibited very low friction (COF = 0.10) in air but very high friction (COF = 0.78) under high vacuum. 

Apparently, low friction in both high-vacuum and atmospheric conditions is unique to the brass/ta-C pair and cannot be obtained with either material alone.

#### 3.2.3. Long-Term Performance Experiments

To study the long-term performance of the brass/ta-C pair, tribological long-term experiments with equivalent sliding distances of 54.0 m, 67.5 m, and 72.0 m instead of 12 m were conducted at pressures of 3.1 × 10^−7^ mbar, 3.6 × 10^−7^ mbar, and 3.3 × 10^−7^ mbar, respectively.

The results from the long-term experiments presented in Table 3 show that the friction coefficient of the brass/ta-C pairing is not stable under high vacuum for longer sliding distances. In two out of the three experiments, the friction coefficient jumped more than 0.5 after an induction period of about 17 m and 30 m (not shown). A similar behavior was observed for the wear rate, with a significant increase of one order of magnitude.

In contrast, the experiments conducted with a sliding distance of 54.0 m exhibited low friction (COF 0.06 up to 0.15) and low wear, similarly to the experiments conducted for 12.0 m of sliding distance. However, the onset of the high-wear and high-friction regime in the 67.5 m and 72 m experiment occurred very early, long before the 54 m sliding distance. Therefore, this onset seems to be rather unstable and can occur over a longer time. 

**Figure 5:**
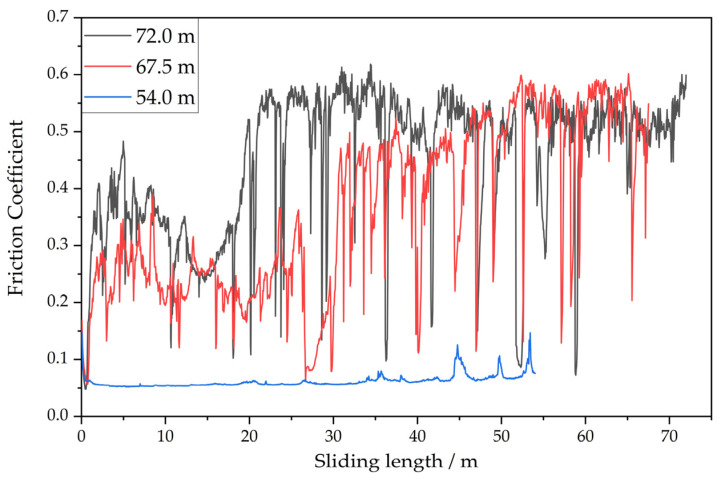
COF for the long-term experiments of the ta-C/brass pairing under high vacuum, with an equivalent sliding distance of 54.0 m, 67.5 m and 72.0 m. The long-term degradation of friction is visible in two experiments.

As visible in Figure 5, the average friction coefficients for the 67.5 m and 72.0 m experiments never reached a stable low-friction phase in the beginning and, moreover, increased sharply after about 30 m and 18 m, respectively, without recovery. 

On the other hand, friction stayed low for the 54.0 m experiment (COF < 0.1), although there was an increasing number of friction spikes in the second half of the experiment. These may indicate the start of similar degradation as with the other two experiments. These results show that the friction performance of brass/ta-C worsens with time.

**Figure 6:**
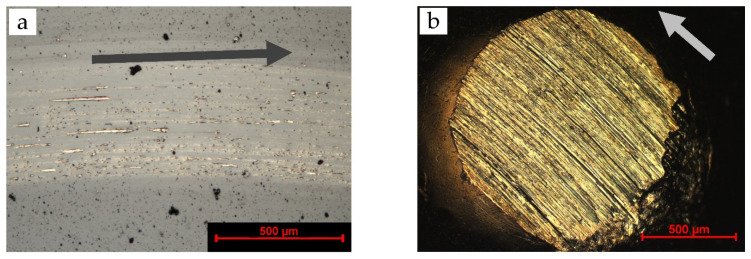
Light microscope images of brass/ta-C sliding experiment under high vacuum showing wear on the (**a**) disc and (**b**) counterbody from the long-term experiment with a sliding distance of 72.0 m. In comparison with the 12.0 m sliding distance, a clear degradation of the disc and counterbody surfaces is visible. The arrows indicate direction of movement of the shown body.

In Figure 6, both the wear track and wear scar of the brass/ta-C system are shown, evaluated for a 72.0 m sliding distance. Considerable wear can be seen on both bodies. On the disc (Figure 6a), many grooves in the direction of movement are visible. The results from multiple contact profilometer measurements indicate a maximum depth of 0.18 µm of the wear track, which is less than the coating thickness of 2.1 µm. The coating is far from being consumed by wear, but exhibits a lighter color, possibly from an increased deposit of brass material into the ta-C coating. 

In contrast to the 12 m experiments (see Table 2), the brass counterbody (Figure 6b) shows grooves typical of abrasion and adhesion. On the right side of the counterbody, the rear side in relation to the grinding direction, some material seems to have been smeared or plastically deformed. No tribolayer is visible.

**Figure 7:**
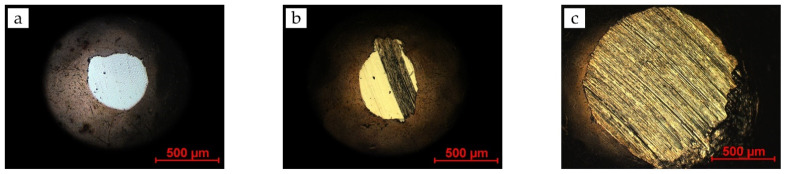
Light microscope images of wear scars on brass counterbodies from brass/ta-C experiments under high vacuum with a sliding length of (**a**) 12.0 m, (**b**) 54.0 m, and (**c**) 72.0 m. Two wear states are visible when comparing the left and right images. The middle image seems to consist of a hybrid between the two states.

In Figure 7, the counterbody wear scar from the 54 m experiment is shown (Figure 7b) alongside the counterbody from the 12 m experiment discussed in Section 3.1 (Figure 7a), as well as the counterbody from the 72 m experiment (Figure 7c). 

The counterbody wear of Figure 7b seems to show two different wear states. Predominantly on the left, the counterbody seems to be worn clean and smooth. It looks very similar to the way the brass counterbody behaved in the counterbody screening (Figure 7a). In essence, this seems to be the wear behavior in the beginning period of the brass/ta-C experiment. On the right of Figure 7b, a streak with a very rough surface is visible. This is reminiscent of the counterbody wear in the 72.0 m long-term experiment (Figure 7c). 

## 4. Discussion

From the results presented in Section 3, it was found that among seven counterbody materials tested against a ta-C disc, brass showed exceptionally low friction (µ < 0.1) under high and medium vacuum, a value commonly found for transition metal dichalcogenides, such as MoS_2_. In contrast, all other tested materials resulted in much higher friction of 0.28 < µ < 0.81, which is typical for vacuum conditions where surface passivation is hindered and cold welding between materials easily occurs. 

In the following section, the interesting nature of the brass/ta-C tribopair will be discussed in detail. 

First, the general wear behavior of the brass/ta-C system can be explained by the low hardness of brass in comparison to that of superhard ta-C. The difference in hardness causes the brass counterbody to wear, whereas the ta-C coating does not show any signs of wear at all. The soft metal acts as sacrificial material.

However, the low hardness and soft metal nature of brass does not provide a simple explanation for the low-friction phenomenon because other soft metals, such as bronze and copper, had much higher friction. Thus, the exceptional smooth wear scar without any sign of transfer layer on the brass counterbody, in addition to the exceptionally low friction, stands out from all other materials. 

A similar smooth surface is seen for the two ceramics. In their case, the high hardness resulted in low wear, but in both cases, a dark transfer layer can been seen on the counterbody wear scar. 

Comparing the brass/ta-C system with brass/steel and steel/ta-C, it was shown that the low friction of brass/ta-C is unique to the combination of the paired materials. Neither substituting steel for brass nor steel for ta-C led to low friction under either ambient or vacuum conditions. Therefore, brass plays a critical role in the special interaction with the ta-C coating, e.g., passivation, which steel cannot provide.

Further study of the brass/ta-C system at higher pressures showed persistent low friction at p = 1 × 10^−2^ mbar (µ = 0.055) and p = 1 × 10^3^ mbar (µ = 0.129). Interestingly, this versatile behavior is based on a different wear mechanism of the brass counterbody, as evidenced by light microscopy and Raman spectroscopy. 

Under high vacuum, the brass counterbody exhibits high wear but has a smooth contact area free of carbon and free of a visible transfer layer. Under medium vacuum, low wear with a stable, carbonaceous transfer layer is found. In atmospheric conditions, wear is also low, and a stable, carbon-free layer is found, possibly caused by oxidation. 

Interestingly, a carbon-containing transfer layer was only formed under medium vacuum but not under high vacuum. Residual gases possibly play a role in either forming or stabilizing the transfer film, which is composed of carbon transfer from the ta-C coating. In atmospheric conditions, the abundance of water and oxygen leads to an easy and carbon-free passivation of the brass counterbody. Under high vacuum, no passivation takes place on the brass counterbody, leading to increased wear. 

However, all of these wear mechanisms result in a low friction surface on the brass counterbody, which can interact with the undisturbed ta-C carbon surface. 

The flexibility to achieve such low friction in a wide range of pressures with corresponding but different passivation mechanisms is unique and has been observed only in the ta-C/brass combination so far. 

The study of the long-term behavior of the brass/ta-C system under high vacuum led to the discovery of a significant friction increase for some cases early during sliding. This transition from low friction to high friction occurred at different times. It was not visible for the 12.0 m experiments and the 54.0 m experiment but occurred very early during the experiments with 67.5 m and 72.0 m sliding length. It must be noted that this increase occurs for the averaged friction coefficient; however, for single short events, friction fell to levels of µ~0.1 again, as if it had “healed”, only to revert to the high-friction level shortly after. 

During the low-friction regime, the contact area remained small, corresponding to a quite high contact pressure of approx. 25 MPa. Once a transition to the high-wear regime had occurred, the counterbody wear scar had a rough surface with deep grooves. The contact area increased to about four times the size, resulting in a contact pressure of approx. 6.25 MPa. In addition, the ta-C disc had a more metallic appearance overall, with some embedded metal particles following the sliding direction. 

The origin of this low friction phenomenon can only be speculated. The ta-C coating itself is passivated in atmospheric conditions but is not known to retain or replenish its passivation when under vacuum due to an absence of water or oxygen [4,6]. If the transition to the high-friction regime were due to the loss of ta-C passivation only, no short fallbacks to the low friction regime would occur. Therefore the low-friction phenomenon must be at least partially attributed to something else. 

Obviously, the increasing roughness in the long-term experiments is associated with the loss of low friction. Therefore, the very smooth brass surface in the beginning must be a key factor for low friction. Accordingly, this kind of smooth surface was not found for the other soft metals that had higher friction. 

Smooth surfaces apart from brass were only found for the two ceramic counterbodies, which were probably retained due to their high hardness and high abrasion resistance. However, SiC had very high friction, presumably due to chemical interaction of the SiC with the carbon from the ta-C coating. A dark-colored transfer layer was also visible. Al_2_O_3_ showed somewhat lower friction, most likely due the combination of its passive state towards carbon and the smooth surface. 

The loss of the low-friction state comes with signs of adhesion wear, where brass material is transferred to the ta-C surface with subsequent cold welding. This process occurs gradually over the contact area, as Figure 7b suggests, and eventually covers the whole contact area. It is unclear why material is not transferred in the beginning but later in the process. 

Two factors are proposed to explain the material transfer. 

First, during sliding and generation of the smooth contact area, brass material is removed and deposited behind the wear scar. Some material might detach and generate particles that are pulled into contact with the next revolution. The particle is crushed and smeared on the carbon coating, resulting in comet-trail-like marks on the ta-C coating (Figure 6a). Due to the high local contact pressure, the passivation of the ta-C disc is lost, so the brass transfer material stays deposited on the carbon coating. Subsequently, a local brass/brass contact is formed. 

As a second aspect, the inner defect structure of the ta-C coating with its growth defects might contribute to this behavior. Even when carefully lapped to a smooth surface, after some tribological loading, loosely bound growth defects might debond from the coating and leave a hole. Such holes might ease material removal from the counterbody or adhesion of brass wear particles for geometrical reasons. They may even provide a place for the brass particles to embed themselves into.

Both processes, generation of loose brass wear particles and generation of holes from growth defects, might happen randomly and would explain the occurrence of such a sudden transition from low friction to high friction at different times during the experiment. 

Considering the observations from all experiments, a strong correlation of a smooth, passive surface and low friction was found. Whereas the ta-C coating exhibits such properties from the beginning, generation of such a stable and smooth surface on the softer counterbody seems to be the unique key feature found only for the brass/ta-C tribopair. 

## 5. Conclusions

Seven different counterbody materials were tested against ta-C coatings under high vacuum (p = 1 × 10^−7^ mbar) in sliding contact regarding their tribological behavior with 12 m sliding distance. The combination of ta-C with brass showed the lowest friction (COF = 0.08). The ta-C coating did not show any measurable wear or structural change, with moderate wear observed on the brass counterbody (8.0 × 10^−6^ mm^3^/(N × m)). Other counterbody materials showed much higher friction and/or wear. 

The observed beneficial properties were found to be unique to the combination of brass/ta-C and could not be reproduced when substituting either the disc or counterbody with steel. This suggests that the low friction results from the generation of a very smooth brass surface, which acts passively towards the ta-C coating under a vacuum. 

Further experiments showed that such low friction behavior of the brass/ta-C system can also be obtained under medium vacuum (p = 1 × 10^−2^ mbar) and atmospheric conditions (p = 1 × 10^3^ mbar). However, different wear and passivation mechanisms were observed on the brass counterbody for each condition. Based on light microscopy and Raman spectroscopy, they were identified as non-carbon tribolayer (atmospheric conditions), carbonaceous tribolayer (medium vacuum), and high-wear polishing (high vacuum). 

For some sliding experiments under high vacuum, a stable and low friction state was not reached initially, and friction increased even further during the experiment. In these cases, wear marks were visible on the ta-C coatings, and high wear and grooves were visible on the brass counterbodies. The rather random and fast transition from low friction, low wear, and smooth counterbody surface to high friction, high wear, and grooved surface indicates a fundamental change in mechanism, possibly from cold welding with transferred brass material. Further study of this transition phenomenon is required to understand the mechanism behind it and, consequently, its circumvention. 

Nonetheless, the studied brass/ta-C system shows unique friction and wear properties in atmospheric conditions and under a vacuum up to p = 1 × 10^−2^ mbar, allowing for the establishment of a stable and versatile low-friction tribosystem for a wide span of pressures. A potential use is in earthbound industrial vacuum applications occasionally exposed to ambient pressure. 

## Figures and Tables

**Table 1:** Counterbody ball materials, vendor, diameter, grade specification, and roughness. The roughness of the counterbodies is calculated from the manufacturer’s grade in Ra (arithmetical mean height of a line).

Counterbody Material	Vendor	Diameter and Grade	Roughness Ra/µm
Brass—CuZn35	TIS Wälzkörpertechnologie Gauting, Germany	10 mm, G200	Ra < 0.203 µm
Silicon carbide SiC	SITUS Technicals Wuppertal, Germany	10 mm, G25	Ra < 0.051 µm
Aluminum oxide Al_2_O_3_	Sd Hartstofftechnik Jestetten, Germany	10 mm, G10	Ra < 0.025 µm
Steel—1.3505	TIS Wälzkörpertechnologie Gauting, Germany	10 mm, G5	Ra < 0.02 µm
Steel coated with ta-C	Fraunhofer IWS Dresden, Germany	10 mm, not specified	Ra ≈ 0.1 µm
Copper	Shandong Yuncheng County Mingliang Steel Ball Factory, Heze, China	10 mm, not specified	not specified
Bronze—CuSn6	Ballcenter Handelsgesellschaft mbH & Co. KG, Neuhof, Germany	9.525 mm, G200	Ra < 0.203 µm

**Table 2:** Brass/ta-C pairs tested at pressures of 10^3^, 10^−2^, and 1.1 × 10^−7^ mbar. Microscopic images of disc and counterbody wear tracks are shown with corresponding Raman signal. Red line shows signal in tribological contact area, and black line shows signal from pristine area outside the contact area. *y*-axis = intensity (a.u.); *x*-axis = Raman shift/cm^−1^.

Pressure	Disc Wear Track	Disc Raman Spectra	Counterbody Wear Scar	Counterbody Raman Spectra
10^3^ mbar	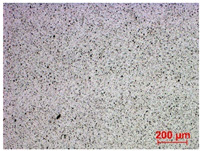	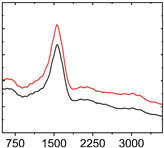	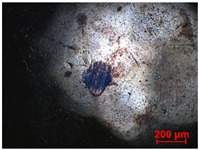	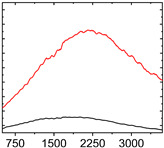
10^−2^ mbar	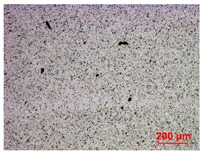	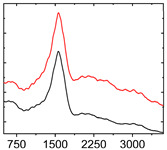	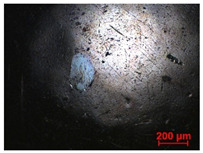	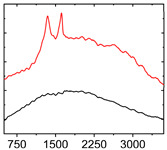
10^−7^ mbar	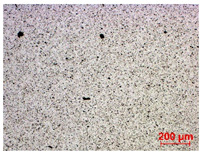	No wear track visible	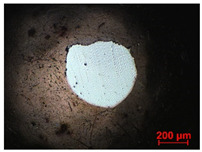	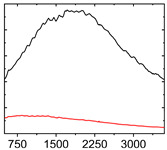

**Table 3:** COF and specific wear rate results from the three long-term experiments of brass/ta-C under high vacuum. In two out of the three experiments, the friction coefficient and wear behavior worsened significantly in the long term. Results for 12.0 m sliding distance are shown for comparison. No wear on the disc of the 12.0 m and 54.0 m brass/ta-C experiments was found; therefore, the minimum measurable wear is reported.

Equivalent Distance	Friction Coefficient Average of Last 5 m	Specific Wear Rate/mm³/Nm	
		Disc	Counterbody
12.0 m	0.08 ± 1.4 × 10^−4^	1 × 10^−6^	8.0 × 10^−6^
54.0 m	0.08 ± 0.02	3 × 10^−7^	4.5 × 10^−6^
67.5 m	0.52 ± 0.07	1.2 × 10^−6^ ± 6.9 × 10^−7^	6.9 × 10^−5^
72.0 m	0.52 ± 0.03	2.9 × 10^−6^ ± 1.2 × 10^−6^	4.6 × 10^−5^

## Data Availability

The raw data can be found at: https://zenodo.org/record/6373784 (accessed on 17 February 2022).
